# Incidence and potential risk factors for remdesivir-associated bradycardia in hospitalized patients with COVID-19: A retrospective cohort study

**DOI:** 10.3389/fphar.2023.1106044

**Published:** 2023-02-01

**Authors:** Yazed Saleh Alsowaida, Fadi Shehadeh, Markos Kalligeros, Eleftherios Mylonakis

**Affiliations:** ^1^ Division of Infectious Diseases, Department of Medicine, The Warren Alpert Medical School, Brown University and Rhode Island Hospital, Providence, RI, United States; ^2^ Department of Clinical Pharmacy, College of Pharmacy, Hail University, Hail, Saudi Arabia; ^3^ School of Electrical and Computer Engineering, National Technical University of Athens, Athens, Greece

**Keywords:** remdesivir (GS-5734), COVID-19, bradycardia, cardiotoxicity, pharmacovigilance

## Abstract

**Background:** Remdesivir is widely used for the management of COVID-19 and several studies have reported bradycardia as a potential side effect associated with this agent. The aim of the present study was to evaluate the incidence rate, severity, and potential risk factors of remdesivir-associated bradycardia.

**Methods:** We performed a retrospective cohort study among hospitalized adult patients with COVID-19 who were treated with remdesivir from March 2020 to October 2021. Our primary outcome of interest was the incidence rate and severity of bradycardia after remdesivir administration. We defined mild bradycardia as a heart rate of 51–59 beats per minute, moderate bradycardia as a heart rate of 41–50 beats per minute, and severe bradycardia as a heart rate of ≤40 beats per minute. We also performed univariable and multivariable regression analyses to determine potential bradycardia risk factors. Baseline characteristics were reported as means with standard deviations or medians with interquartile ranges (IQRs). All the statistical tests are shown as odds ratios (ORs) with 95% confidence intervals (CIs).

**Results:** In total, 1,635 patients were included in this study. The median age with IQR was 68 (57–79) years and 51.7% of the patients were male. In total, 606 (37.1%) patients developed bradycardia. Among them, 437 patients (26.7%) developed mild bradycardia, 158 patients (9.7%) moderate bradycardia, while 11 patients (0.7%) experienced severe bradycardia. In our adjusted multivariate logistic regression, the odds of bradycardia development after remdesivir administration were higher among patients with age ≥65 years (OR 1.76, 95% CI: 1.04–2.99, *p* = 0.04), those with hypertension (OR 1.37, 95% CI: 1.07–1.75, *p* = 0.01), and obesity (OR 1.32, 95% CI: 1.02–1.68, *p* = 0.03).

**Conclusion:** More than 1 out of 3 patients (37%) who received remdesivir for COVID-19 developed bradycardia with the majority of these patients developing mild or moderate bradycardia that is usually a benign manifestation not needing treatment in most cases. Age ≥65 years, hypertension, and obesity were potential risk factors for remdesivir-associated bradycardia among hospitalized COVID-19 patients. Clinicians should be aware of this adverse event and consider close clinical monitoring for patients at high risk for this adverse event.

## 1 Introduction

The development of effective drugs for the treatment and prevention of COVID-19 has been a global priority (Beigel et al., 2020). Numerous drugs have been evaluated in clinical trials for COVID-19, for example, hydroxychloroquine, antiviral drugs, glucocorticoids, and neutralizing antibodies (Bhimraj et al., 2022; Thakur et al., 2022). However, only limited drugs have shown benefits in COVID-19 and are recommended for use by the Infectious Diseases Society of America. Since COVID-19 affects multiple organ systems and is associated with neurological and cardiovascular manifestations, several observational studies have been conducted to (Kang et al., 2020; Sharma et al., 2021) observational studies evaluated if there are mortality benefits of aspirin and angiotensin-converting enzyme inhibitors/angiotensin II receptor blockers (Srivastava and Kumar, 2021; Azad and Kumar, 2022). However, the evidence is inconclusive to recommend these therapies for COVID-19 patients.

Remdesivir is an antiviral agent with a broad spectrum of activity against several viruses that include SARS-CoV-1, SARS-CoV-2, MERS-CoV, and *Filoviridae* viruses (e.g., Ebola virus and Marburg virus) (Jorgensen et al., 2020; Gilead, 2022). Currently, remdesivir is approved by the Food and Drug Administration for the treatment of COVID-19 in adult and pediatric patients (Gilead, 2022). The World Health Organization, the Infectious Diseases Society of America, and the National Institute of Health recommend using remdesivir for ambulatory or hospitalized patients with mild-to-moderate COVID-19 at high risk for progression to severe disease and hospitalized patients on supplemental oxygen (Bhimraj; National Institute of Health, 2022; World Health Organization).

Generally, remdesivir is well-tolerated and the most common adverse drug reactions are nausea and transaminase elevations (Jorgensen et al., 2020). Several post-marketing studies have reported marked bradycardia with the administration of remdesivir (Attena et al., 2021; Brunetti et al., 2021; Singh and Kamath, 2021; Touafchia et al., 2021). Pharmacologically, remdesivir is a monophosphoramide nucleoside prodrug that undergoes intracellular metabolism to its active form as remdesivir triphosphate (Jorgensen et al., 2020). When activated, remdesivir is incorporated into the viral RNA by RNA-dependent RNA-polymerase, which terminates the RNA chain and inhibits viral replication. Notably, the active metabolite of remdesivir is an adenosine triphosphate analog (Nabati and Parsaee, 2022). Adenosine triphosphate has negative chronotropic and dromotropic actions by suppressing cardiac pacemakers firing at the sinoatrial node and the cardiac conduction at the atrioventricular node (Pelleg and Belhassen, 2010). Therefore, the adenosine triphosphate cardiac effects mediated by remdesivir triphosphate could play a role in the development of remdesivir-associated bradycardia.

Bradycardia can be associated with adverse health outcomes that may lead to impaired functional capacity and provocation of cardiac arrhythmias (Kusumoto et al., 2019). The reported incidence rates of bradycardia associated with remdesivir are inconclusive and vary from 16.8%–49% (Bistrovic et al., 2022; Schreiber et al., 2022). In this study, we aim to evaluate the incidence rate, severity, and potential risk factors for the development of bradycardia among hospitalized patients with COVID-19 who received remdesivir.

## 2 Methods and methods

### 2.1 Study setting and design

We performed a retrospective cohort study for patients admitted at two quaternary care hospitals, namely Rhode Island Hospital and the Miriam Hospital, located in Providence, Rhode Island, United States. Patients were identified using electronic health records from March 2020 to October 2021. The project was approved by Lifespan Institutional Review Board (Protocol# 1589781).

### 2.2 Eligibility criteria

Hospitalized adult patients (≥18 years old) with a positive SARS-CoV-2 test, who received at least one dose of remdesivir were considered eligible for inclusion. Patients who were not treated with remdesivir were excluded. Remdesivir was administered intravenously with a loading dose of 200 mg on day 1 then followed by a maintenance dose of 100 mg daily for 2–10 days (Jorgensen et al., 2020). To be considered remdesivir-associated, bradycardia development must occur within 24 h after remdesivir administration to account for the half-life of the drug and its active metabolite (Jorgensen et al., 2020).

Our primary outcome of interest was the incidence rate and severity of bradycardia after remdesivir administration. We defined bradycardia as a heart rate of <60 beats per minute (Kusumoto et al., 2019). We evaluated the outlier heart rate readings for validation and excluded erroneous heart rate readings based on patients’ heart rate trends. We defined the severity of bradycardia as follows: mild bradycardia (heart rate 51–59 beats per minute), moderate bradycardia (heart rate 41–50 beats per minute), and severe bradycardia (heart rate ≤40 beats per minute). As a secondary outcome, we examined potential risk factors that could be associated with bradycardia development in the setting of remdesivir use.

### 2.3 Data collection

For each patient, the following data were extracted: age, sex, race, ethnicity, weight, vital signs, laboratory values, preexisting medical conditions, and intensive care unit admission. We collected these measurements once at the initiation of remdesivir therapy.

### 2.4 Statistical analysis

The baseline characteristics were reported as means with standard deviations or medians with IQRs and were analyzed statistically using the Student’s *t*-test and the Mann-Whitney-Wilcoxon test, respectively. Categorical data were compared using Pearson’s chi-square test.

Outcomes were analyzed using univariate and multivariate logistic regression. In the multivariate logistic regression, we adjusted the model for potential confounders. Specifically, we adjusted for age, gender, race, intensive care unit admission, baseline oxygen requirements, hypertension, diabetes mellitus, heart diseases (congestive heart failure and cardiac arrhythmias), presence of baseline bradycardia, obesity, and hypothyroidism. Moreover, we adjusted for drugs that may cause arrhythmia or alter heart rate measurements including beta-blockers, calcium channel blockers, adrenergic drugs, anti-arrhythmic drugs, macrolide antibiotics, azoles antifungal agents, fluoroquinolone antibiotics, serotonin or norepinephrine reuptake inhibitors, and tricyclic antidepressants. Lastly, to adjust for the severity of illness on admission, we calculated the National Early Warning Score (NEWS) (Uppanisakorn et al., 2018).

Statistical significance was set at a *p*-value threshold of 0.05, and all analytical tests were performed using Stata, version 17 (Stata Corporation, College Station, TX, USA).

## 3 Results

### 3.1 Baseline characteristics

In total 1,635 patients were included in the study. The median age was 68 years (IQR: 57–79) with 845 patients (51.7%) being males. Furthermore, 1,069/1,635 (65.4%) patients were Non-Hispanic White, 295/1,635 (18%) patients were Hispanic or Latino, and 155/1,635 (9.5%) patients were Non-Hispanic Black. The most common comorbidities were hypertension (986/1,635; 60.3%), diabetes mellitus (509/1,635; 31.1%), heart disease (414/1,635; 25.3%), and chronic pulmonary disease (379/1,635; 23.2%). Furthermore, age ≥65 years, intensive care unit admission, hypertension, and obesity were more common among patients who developed bradycardia after remdesivir administration. Complete information on patients’ baseline characteristics is available in Table 1.

**TABLE 1:** Baseline characteristics of the included patients.

	Total N = 1,635	Normal heart rate N = 1,029	Developed bradycardia N = 606	*p*-value
**Patient age, years (IQR)**	68.00 (57.00–79.00)	67.00 (55.00–78.00)	70.00 (60.00–81.00)	**<0.001**
**Patient sex**				0.46
**Female**	790 (48.32%)	490 (47.62%)	300 (49.50%)	
**Male**	845 (51.68%)	539 (52.38%)	306 (50.50%)	
**Race**				0.11
**Non-Hispanic White**	1,069 (65.38%)	651 (63.27%)	418 (68.98%)	
**Hispanic or Latino**	295 (18.04%)	196 (19.05%)	99 (16.34%)	
**Non-Hispanic Black**	155 (9.48%)	107 (10.40%)	48 (7.92%)	
**Other/unknown**	116 (7.09%)	75 (7.29%)	41 (6.77%)	
**Intensive care unit admission**				**<0.001**
**No**	1,336 (81.71%)	880 (85.52%)	456 (75.25%)	
**Yes**	299 (18.29%)	149 (14.48%)	150 (24.75%)	
**Baseline oxygen requirements**				**0.02**
**Ventilator**	3 (0.2%)	1 (0.1%)	2 (0.3%)	
**High flow/NIPPV**	160 (9.8%)	91 (8.8%)	69 (11.4%)	
**Low flow**	977 (59.8%)	600 (58.3%)	377 (62.2%)	
**Room air**	495 (30.3%)	337 (32.8%)	158 (26.1%)	
**Minimum heart rate, beats per minute (IQR)**	63.00 (55.00–71.00)	69.00 (64.00–76.00)	53.00 (50.00–56.00)	**<0.001**
Presence of **b**aseline bradycardia	88 (5.4%)	29 (2.8%)	59 (9.7%)	**<0.001**
**Severity of bradycardia**				–
**Mild bradycardia (heart rate 51–59 beats per minute)**	**–**	**–**	437 (26.7%)	
**Moderate bradycardia (heart rate 41–50 beats per minute)**	**–**	**–**	158 (9.7%)	
**Severe bradycardia (heart rate ≤ 40 beats per minute)**	**–**	**–**	11 (0.7%)	
**Heart disease**	414 (25.32%)	252 (24.49%)	162 (26.73%)	0.31
**Pulmonary circulation disorders**	83 (5.08%)	48 (4.66%)	35 (5.78%)	0.32
**Peripheral vascular disorders**	126 (7.71%)	76 (7.39%)	50 (8.25%)	0.53
**Hypertension**	986 (60.31%)	584 (56.75%)	402 (66.34%)	**<0.001**
**Paralysis**	6 (0.37%)	4 (0.39%)	2 (0.33%)	0.85
**Other neurological disorders**	135 (8.32%)	90 (8.75%)	45 (7.43%)	0.35
**Chronic pulmonary disease**	379 (23.18%)	238 (23.13%)	141 (23.27%)	0.95
**Diabetes mellitus**	509 (31.13%)	329 (31.97%)	180 (29.70%)	0.34
**Hypothyroidism**	147 (8.99%)	83 (8.07%)	64 (10.56%)	0.09
**Renal failure**	171 (10.46%)	101 (9.82%)	70 (11.55%)	0.27
**Liver disease**	64 (3.91%)	42 (4.08%)	22 (3.63%)	0.65
**Peptic ulcer disease excluding bleeding**	22 (1.35%)	12 (1.17%)	10 (1.65%)	0.41
**AIDS/HIV**	8 (0.49%)	6 (0.58%)	2 (0.33%)	0.48
**Lymphoma**	26 (1.59%)	18 (1.75%)	8 (1.32%)	0.50
**Metastatic cancer**	55 (3.36%)	40 (3.89%)	15 (2.48%)	0.13
**Solid tumor without metastasis**	171 (10.46%)	105 (10.20%)	66 (10.89%)	0.66
**Rheumatoid arthritis/collaged vascular disease**	71 (4.34%)	46 (4.47%)	25 (4.13%)	0.74
**Coagulopathy**	78 (4.77%)	46 (4.47%)	32 (5.28%)	0.46
**Obesity**	450 (27.52%)	261 (25.36%)	189 (31.19%)	**0.01**
**Weight loss**	11 (0.67%)	6 (0.58%)	5 (0.83%)	0.56
**Fluid and electrolyte disorders**	209 (12.78%)	135 (13.12%)	74 (12.21%)	0.60
**Blood loss anemia**	20 (1.22%)	15 (1.46%)	5 (0.83%)	0.26
**Deficiency anemia**	61 (3.73%)	36 (3.50%)	25 (4.13%)	0.52
**Alcohol abuse**	35 (2.14%)	17 (1.65%)	18 (2.97%)	0.08
**Drug abuse**	27 (1.65%)	21 (2.04%)	6 (0.99%)	0.11
**Psychoses**	30 (1.83%)	19 (1.85%)	11 (1.82%)	0.96
**Depression**	297 (18.17%)	178 (17.30%)	119 (19.64%)	0.24
**NEWS**				0.75
**NEWS = 0**	116 (7.09%)	78 (7.58%)	38 (6.27%)	
**Low Severity (NEWS 1–4)**	791 (48.38%)	492 (47.81%)	299 (49.34%)	
**Medium Severity (NEWS 5–6)**	438 (26.79%)	278 (27.02%)	160 (26.40%)	
**High Severity (NEWS ≥7)**	290 (17.74%)	181 (17.59%)	109 (17.99%)	
**Drugs that interfere with heart rate**	1,443 (88.26%)	907 (88.14%)	536 (88.45%)	0.85

HIV/AIDS, human immunodeficiency virus/acquired immunodeficiency syndrome; IQR, interquartile range; NIPPV, Nasal intermittent positive pressure ventilation, and NEWS: National Early Warning Score. Bolded *p*-values are statistically significant.

### 3.2 Incidence rate and severity of bradycardia with remdesivir

**FIGURE 1:**
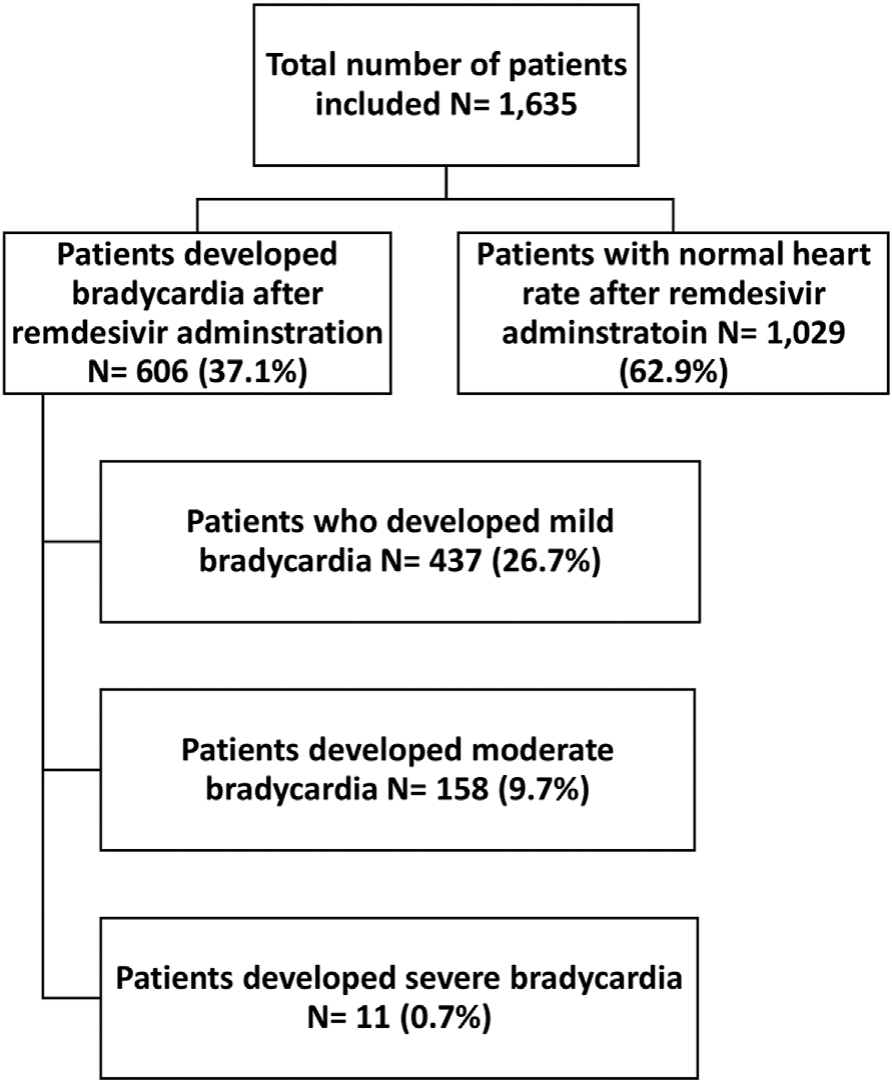
Incidence and severity of remdesivir-associated bradycardia.

Among 1,635 patients who received remdesivir, 606 patients (37.1%) developed bradycardia. More specifically, 437 patients (26.7%) developed mild bradycardia, 158 patients (9.7%) moderate bradycardia, and 11 patients (0.7%) developed severe bradycardia, Figure 1.

### 3.3 Potential risk factors for remdesivir-associated bradycardia

After adjusting for confounders in our multivariate logistic regression, bradycardia development after remdesivir administration was associated with age ≥65 years (OR 1.76, 95% CI: 1.04–2.99, *p* = 0.04), hypertension (OR 1.37, 95% CI: 1.07–1.75, *p* = 0.01), and obesity (OR 1.32, 95% CI: 1.02–1.69, *p* = 0.03). Diabetes mellitus was negatively associated with bradycardia after remdesivir administration (OR 0.71, 95% CI: 0.56–0.91, *p* = 0.01). Heart disease (OR 0.86, 95% CI: 0.66–1.11, *p* = 0.25), drugs that interfere with heart rate (OR 0.89, 95% CI: 0.64–1.23, *p* = 0.49), hypothyroidism (OR 1.11, 95% CI: 0.77–1.60, *p* = 0.56), NEWS (OR 0.97, 95% CI: 0.92–1.02, *p* = 0.26), and male gender (OR 0.92, 95% CI: 0.74–1.13, *p* = 0.41) were not associated with bradycardia after remdesivir administration. Complete information for the multivariate logistic regression is available in Table 2.

**TABLE 2:** Multivariate analysis of factors associated with bradycardia.

Variable	Odds ratio	95% confidence interval		*p*-value
**Age**				
**40–65 years old**	1.51	0.89	2.56	0.12
**Age ≥65 years and older**	1.76	1.04	2.99	**0.04**
**Male gender**	0.92	0.74	1.13	0.41
Presence of baseline bradycardia	3.64	2.27	5.83	**<0.001**
**Baseline oxygen requirements**				
**Low flow**	1.08	0.74	1.57	0.69
**Ventilator**	3.13	0.27	36.15	0.36
**Room air**	0.78	0.50	1.24	0.29
**Race**				
**Hispanic or Latino**	0.89	0.67	1.18	0.41
**Non-Hispanic Black**	0.71	0.49	1.04	0.08
**Other/unknown**	0.92	0.60	1.41	0.71
**Intensive care unit admission**	1.94	1.46	2.58	**<0.001**
**Drugs that interfere with heart rate**	0.89	0.64	1.23	0.49
**Hypertension**	1.37	1.07	1.75	**0.01**
**Obesity**	1.32	1.02	1.69	**0.03**
**Hypothyroidism**	1.11	0.77	1.60	0.56
**Diabetes mellitus**	0.71	0.56	0.91	**0.01**
**Heart disease**	0.86	0.66	1.11	0.25
**NEWS**	0.97	0.92	1.02	0.26

NEWS, National Early Warning Score. Bolded *p*-values are statistically significant.

### 3.4 Characteristics and outcomes of patients with severe bradycardia

Eleven patients in our study developed severe bradycardia. Their median age was 72 (IQR: 60–85) and 8 patients (73%) were males. The most common comorbidities were hypertension (7/11; 64%), heart disease (6/11; 55%), and chronic pulmonary disease (4/11; 35%). Despite bradycardia, all patients completed their remdesivir therapy, and their heart rate was closely monitored. Among the 11 patients who developed severe bradycardia, 3 patients had documented complications related to bradycardia after remdesivir administration and the cases are presented in detail below. Specifically, one patient received a dose of atropine 0.1 mg injection to reverse bradycardia, 1 patient had a syncopal episode, and 1 patient had an emergency medical response due to severe bradycardia. Notably, none of those patients developed atrioventricular block or sick sinus syndrome. In all the cases, the medical teams did not identify the possible reason behind bradycardia development.

### 3.5 Patients who developed complications due to bradycardia

Patient #1 was a 71-year-old male with a past medical history of chronic pulmonary disease, coronary artery disease, type I diabetes, hypertension, hypothyroidism, and decompensated alcoholic cirrhosis with Model for End-Stage Liver Disease score of 9. He was admitted to the hospital for respiratory distress with a blood oxygen saturation of 83% and tachycardia of 120 beats per minute due to COVID-19. He was admitted to the intensive care unit and placed on bilevel-positive airway pressure. The medical team decided to initiate remdesivir and dexamethasone. The patient had fluctuating bradycardia since the second day of remdesivir therapy. On the last day of remdesivir therapy, he had two episodes of bradycardia of 38 and 40 beats per minute for which he received an atropine 0.1 mg injection. He had no additional bradycardia episodes after the atropine dose. Ultimately, 5 days after completion of remdesivir therapy, the patient died of respiratory failure secondary to COVID-19 and aspiration pneumonia.

Patient #2 was a 93-year-old female with a past medical history of Alzheimer’s disease, essential tremor, and osteoarthritis. She presented to the hospital with respiratory distress and was found to have COVID-19. She was treated with 5 days course of remdesivir in combination with a 10-day course of dexamethasone. On day 3 of remdesivir administration, she developed bradycardia with a heart rate in 30–40 s beats per minute. Her bradycardia persisted for the duration of her 5-day remdesivir therapy while she also experienced a syncopal episode. Once remdesivir therapy was completed, her heart rate normalized. The medical team was unable to identify any other causes of bradycardia. The patient improved and was discharged to a skilled nursing facility with supplementary oxygen.

Patient #3 was a 68-year-old female with a past medical history of atrial fibrillation and hypertension. She presented to the hospital for cough, fatigue, and shortness of breath due to COVID-19. She was started with remdesivir and dexamethasone. On day 1 of remdesivir administration, she became bradycardic with heart rate in the 30 s–40 s beats per minute and had multiple 3.4–4 s pause on cardiac telemetry warranting an emergency medical response. The medical team administered calcium gluconate on the first day of remdesivir therapy to stabilize the myocardium from hypocalcemia (level was 8 mg/dL) and counteract cardiotoxicity from chronic diltiazem use. Ultimately, her heart rate spontaneously normalized, and she did not experience further bradycardia episodes and she completed her 5-day course of remdesivir therapy. The medical team was unable to identify any other cause of bradycardia. The patient eventually improved, and she was weaned off the oxygen therapy and discharged home.

## 4 Discussion

Remdesivir is an important therapeutic option for the treatment of certain patients with COVID-19 (Beigel et al., 2020). In the present study, we evaluated the incidence rate, the severity of bradycardia, and the potential risk factors for remdesivir-associated bradycardia in hospitalized patients with COVID-19. The incidence rate of remdesivir-associated bradycardia was 37.1% with the majority of patients (437/1,635; 26.7%) experiencing mild bradycardia. We found that age ≥65 years old, hypertension, and obesity were associated with bradycardia after remdesivir administration.

The incidence rate of remdesivir-associated bradycardia in our study was 37.1%. A study by Pantazopoulos et al. included 118 patients who received remdesivir for COVID-19 treatment and found the incidence rate to be bradycardia after remdesivir administration was 40.6% (Pantazopoulos et al., 2022). Another study by Schreiber et al. (Schreiber et al., 2022) evaluated 375 patients who experienced bradycardia after remdesivir administration for the treatment of COVID-19 and found the incidence rate to be 49%. Compared to our study, a likely explanation for the higher incidence rate of bradycardia with remdesivir in Schreiber et al. (Schreiber et al., 2022) is that they accounted for bradycardia episodes from remdesivir initiation and up to 5 days after remdesivir therapy. Notably, the half-life of remdesivir and its active metabolites is approximately 24 h (Jorgensen et al., 2020). Therefore, that study could have included more episodes of bradycardia due to COVID-19 cardiac complications (Kang et al., 2020).

Moreover, Pallotto et al. (Pallotto et al., 2021) evaluated 62 patients who received remdesivir for COVID-19 treatment and found the incidence rate of remdesivir-associated bradycardia to be 46.8%. Notably, the authors, which defined bradycardia as a heart rate <60 beats per minute in two consecutive measurements or a heart rate <50 beats per minute once which could explain the difference in the incidence rate of remdesivir-associated bradycardia compared to our study. Other studies (Attena et al., 2021; Bistrovic et al., 2022) reported lower incidence rates of bradycardia with remdesivir therapy. Attena et al. (Attena et al., 2021) included 100 patients who received remdesivir for the treatment of COVID-19 and found the incidence rate of remdesivir-associated bradycardia to be 21%. Bistrovic et al. (Bistrovic et al., 2022) evaluated 437 patients who received remdesivir for COVID-19 treatment and found the incidence rate of bradycardia after remdesivir administration was 16.8%. Variations in the incidence rates of remdesivir-associated bradycardia among these studies related to sample size, definitions of bradycardia, differences in baseline characteristics, and comorbidities profiles.

Among patients who developed bradycardia, we found that the majority of them developed mild to moderate bradycardia. Our findings are consistent with other studies that evaluated the severity of bradycardia among COVID-19 patients treated with remdesivir. The study by Attena et al. (Attena et al., 2021) found that among the 21 patients who developed bradycardia after remdesivir administration, 17 (81%) patients had mild to moderate bradycardia and only 4 (19%) patients had severe bradycardia. Also, Pantazopoulos et al. (Pantazopoulos et al., 2022) found the incidence rate of severe bradycardia associated with remdesivir was 7.5% and among those patients, one had QT interval prolongation and two patients had first-degree atrioventricular block. Our evaluation of patients with severe bradycardia revealed that the bradycardia started within 1–2 days of remdesivir initiation and resolved approximately 1 day after discontinuation of remdesivir which corresponds to the half-life of remdesivir and its active metabolites (Jorgensen et al., 2020). Our findings were consistent with the study by (Attena et al., 2021) which revealed that bradycardia is reversible once remdesivir is discontinued. Furthermore, we found that some patients with severe bradycardia may require the administration of atropine and initiation of medical emergency response.

Patients with age ≥65 years old were more likely to develop bradycardia after remdesivir administration. We also identified hypertension as a potential risk factor for remdesivir-associated bradycardia. Age-related cardiovascular changes particularly in the conduction system are plausible explanations for remdesivir-associated bradycardia in patients with age ≥65 years and those with hypertension (Strait and Lakatta, 2012). Our findings expand the results of other studies (Attena et al., 2021; Pallotto et al., 2021) that found a higher frequency of bradycardia among older adults (Attena et al., 2021; Pallotto et al., 2021) and those with hypertension (Attena et al., 2021), although these studies did not adjust for confounders. Furthermore, hypertension is associated with electrocardiographic changes that may be associated with bradycardia, yet further research is needed to explain the underlying pathophysiology (Bird et al., 2020).

Additionally, we found intensive care unit admission as a potential risk factor for remdesivir-associated bradycardia. Critical illness with SARS-CoV-2 is associated with poor outcomes and a high mortality rate because critical illness disturbs multiple organ systems including the heart function which makes patients on remdesivir therapy more vulnerable to bradycardia (Dessie and Zewotir, 2021; Gao et al., 2021). A study by Tajarernmuang et al. (Tajarernmuang et al., 2022) evaluated bradycardia and heart rate fluctuation in critically ill COVID-19 patients, and they found 71.1% (62/86) of the patient had bradycardia and those patients had longer intensive care unit length of stay.

In our study, obesity was also a potential risk factor for bradycardia after remdesivir administration (OR 1.32, 95% CI: 1.02–1.69, *p* = 0.03). Although obesity is a well-established risk factor for cardiovascular diseases, the underlying mechanism for bradycardia in patients with obesity after remdesivir administration is unclear. The underlying mechanism could be attributed to an imbalance between the sympathetic and parasympathetic nervous systems which leads to a higher heart rate variability in patients with obesity (Poirier et al., 2006).

Interestingly, we found that diabetes mellitus was negatively associated with bradycardia development after remdesivir administration (OR 0.71, 95% CI: 0.56–0.91, *p* = 0.01). The relationship between diabetes mellitus and cardiovascular diseases is a well-established and multifactorial (Grisanti, 2018). Apparently, diabetes mellitus had deleterious effects on the heart including autonomic dysfunction and cardiac remodeling. These effects may lead to higher baseline heart rates in patients with diabetes mellitus. ONTARGET/TRANSCEND clinical trials (Bohm et al., 2020) assessed the resting heart rate and the development of cardiovascular outcomes among patients with and without diabetes mellitus aged 55 years or older. The trial included 11,487 patients with diabetes mellitus and 19,450 patients without diabetes. Compared to patients without diabetes mellitus, patients with diabetes mellitus had a statistically significantly higher mean resting heart rate (71.8 ± 9.0 vs. 67.9 ± 8.8, *p* < 0.0001). Thus, the higher heart rate of patients with diabetes mellitus may explain why diabetes mellitus was negatively associated with bradycardia after remdesivir administration. Additional research is needed to elucidate the effects of diabetes mellitus on heart rate after remdesivir administration.

Regarding limitations, our study is observational and retrospective in nature and there is a risk of bias and incomplete or missing data. Although we evaluated the association of remdesivir with bradycardia and its potential risk factors we could not assess causality. This is particularly important because COVID-19 is associated with a wide range of cardiac complications such as arrhythmias and cardiogenic shock that may worsen health outcomes and could cause death due to direct viral cardiotoxicity and excessive inflammation (Kang et al., 2020; Abbasi, 2022). To minimize confounding and evaluate the association accurately, we evaluated bradycardia episodes from remdesivir initiation to 24 h after remdesivir therapy to account for the half-life of remdesivir and its active metabolites (Jorgensen et al., 2020). During the study period, remdesivir was not recommended for ambulatory or pediatric COVID-19 patients; thus, such patients were not included in our study. Additionally, our cohort included patients with comorbidities or concurrent drugs that could have interfered with heart rate; however, we attempted to minimize bias by adjusting for selected confounders in our multivariate logistic regression. We also adjusted for the severity of COVID-19 on admission with NEWS (Uppanisakorn et al., 2018) and baseline oxygen requirements. Lastly, we could not evaluate remdesivir-associated atrioventricular block in patients with mild-moderate bradycardia.

In conclusion, remdesivir is an important antiviral agent for the management of COVID-19. The incidence rate of remdesivir-associated bradycardia in our study was 37.1% with the majority of these patients developing only mild to moderate bradycardia which is usually a benign manifestation not needing treatment in most cases unless atrioventricular block or sick sinus syndrome are present. Age ≥65 years, hypertension, and obesity were potential risk factors for remdesivir-associated bradycardia among hospitalized COVID-19 patients. Our study informs clinicians about the characteristics of patients who are vulnerable to remdesivir-associated bradycardia and close heart rate monitoring for those patients is warranted. Future studies should evaluate the clinical outcomes of remdesivir-associated bradycardia, compare patients who received remdesivir with a cohort of patients with similar characteristics that did not receive remdesivir, and conduct the study over a long-term period.

## Data Availability

The raw data supporting the conclusion of this article will be made available by the authors, without undue reservation.

## Abbreviations

COVID-19, coronavirus disease-2019; CI, confidence interval; IQR, interquartile range; NEWS, national early warning score; OR, odds ratio.
